# The Dignity of Kangal Fish in the Context of the Swiss Animal Protection Act

**DOI:** 10.3390/ani10040561

**Published:** 2020-03-27

**Authors:** Katharina Friedli, Heinrich Binder

**Affiliations:** 1 Tänikon Centre of Anmimal Welfare, 8356 Ettenhausen, Switzerland; katharina.friedli@agroscope.admin.ch; 2 Federal Food Safety and Veterinary Office, 3003 Berne, Switzerland

**Keywords:** Swiss Animal Protection Act, dignity of the animal, weighing of interests, seven-step model, kangal fish

## Abstract

**Simple Summary:**

Since 2008, the Swiss Animal Protection Act protects not only the welfare but also the dignity of animals. This article explains the concept of the dignity of animals in the context of the Act. Weighing of interests is applied in a specific situation to answer the question of whether the dignity of the animal is respected or not. With this method, the strain imposed on the animal and the interests involved in the situation are evaluated and compared. The seven-step model for the weighing of interests is explained in detail. How the weighing of interests deals with practical questions is shown with the example of kangal fish used for therapeutic or recreational purposes.

**Abstract:**

Since 2008, the Swiss Animal Protection Act (AniPA) protects not only the welfare but also the dignity of the animal. Weighing of interests plays a crucial role in the implementation of the dignity concept. This article outlines the concept of the dignity of animals and explains the method of weighing of interests in the context of the AniPA. The ‘Dignity of the Animal’ study group (DSG) of the Federal Food Safety and Veterinary Office (FSVO) has disputed the implementation of this novel concept in animal welfare and has developed a model procedure to ensure that weighing of interests in the context of the AniPA is carried out correctly and in a standardized way. Weighing of interests is performed in seven steps: 1. Describing the aim; 2. presentation of the facts; 3. assessing suitability; 4. assessing necessity; 5. identifying and assessing strain; 6. identifying and assessing interests; and 7. comparing strain vs. interests. The application of the model is shown with the example of kangal fish used for therapeutic or recreational purposes. It shows how the outcome of the weighing of interests will differ for the same setup depending on the nature of interests involved.

## 1. Introduction

In 2008, a revised version of the Swiss Animal Protection Act (AniPA) [1] entered into force. Unlike its predecessor, it protects not only the welfare but also the dignity of the animal. The amendment was considered essential after the term ‘dignity of living beings’ had been added to the Swiss Federal Constitution in 1992, mandating the confederation to legislate on this issue. But the concept of the dignity of the animal remains a controversial issue even after it has been taken into account in the Swiss animal welfare legislation. Philosophers, ethicists, theologians, and jurists still argue about the meaningful use of the term ‘dignity of the animal’ and how it could be defined. However, within the framework of the AniPA, a definition of the term ‘dignity of the animal’ is given in Article 3 letter a. It reads as follows:

*“Inherent worth of the animal that has to be respected when dealing with it. If any strain imposed on the animal cannot be justified by overriding interests, this constitutes a disregard for the animal’s dignity. Strain is deemed to be present in particular if pain, suffering or harm is inflicted on the animal, if it is exposed to anxiety or humiliation, if there is major interference with its appearance or its abilities or if it is excessively instrumentalised.”*

In contrast to the dignity of man, this definition does not guarantee that the dignity of the animal takes priority under all circumstances. To make an assessment in a specific case, the severity of the strain imposed on the animal has to be weighed against possibly overriding interests.

Putting the concept of the ‘dignity of the animal’ into practice in the context of the AniPA has proven to be difficult for several reasons. The definition of ‘dignity of the animal’ according to Article 3 letter a of the AniPA includes several terms which label the strains that have to be considered when dealing with a specific case. However, the meaning of at least some of these terms is not very clear. As far as the possibly overriding interests are concerned, the AniPA does not give any indication on what kind of interests could or should be taken into account (except in the context of animal experiments). Finally, the AniPA lacks information on how the weighing of interests should be done. The ‘Dignity of the animal’ study group (DSG) of the Federal Food Safety and Veterinary Office (FSVO) has disputed these problems and has come up with a model procedure to ensure that weighing of interests in the context of the AniPA is carried out correctly and in a standardized way.

In this article, we will explain how the concept of the dignity of the animal is understood in the AniPA. Moreover, we will show how the weighing of interests can be done. Finally, we will show how the weighing of interests works on a specific case, namely the use of kangal fish (Garra rufa, doctor fish) in two different settings.

## 2. Dignity of the Animal in the Swiss Animal Welfare Legislation

The ‘dignity of living beings’ is a concept from the Swiss Federal Constitution (Article 120) [2] which has been enshrined in national law. Two Acts are relevant in this respect: the Act on Non-Human Gene Technology Engineering (n-HGTE) [3] and the AniPA.

The concept of ‘dignity of living beings’ relates to individual living beings: each individual animal and plant must be treated with dignity. The concept serves not to protect the species in the sense of a group of individuals.

Even though the ‘dignity of living beings’ was included in the Constitution in the context of gene technology, it applies irrespective of how a living being was created, i.e., it applies not only to transgenic animals and plants. It is a general constitutional principle that must be taken into consideration in all areas.

In animal welfare legislation, dignity concerns all areas addressed by the legislation. This is clear from the purpose set out in Article 1 of the AniPA: “The purpose of this Act is to protect the dignity and welfare of animals”.

According to prevailing doctrine, the concept of dignity of living beings is based on a particular eco-ethical position known as hierarchical biocentrism. According to this position, all living beings, i.e., not just human beings or other sentient beings, have inherent worth and should therefore be given moral consideration for their own sake, not simply because they are economically useful or aesthetically valuable to us. This inherent worth is based on what is termed ‘inherent good’. The Constitution assumes that all plants and animals, as well as some other organisms, have an inherent good. In the n-HGTE and the AniPA, however, the term ‘dignity of living beings’ or ‘dignity of the animal’ is confined to specific organisms: plants and animals in the n-HGTE; vertebrates in the AniPA.

Article 8 of the n-HGTE provides that the dignity of living beings must not be disregarded and specifies circumstances in which this statutory provision is not respected:

*“In animals and plants, modification of the genetic material by gene technology must not violate the dignity of living beings. In particular, violation is deemed to have occurred if such modification substantially harms species-specific properties, functions or habits, unless this is justified by overriding legitimate interests.”*

The dignity of living beings/dignity of the animal does not imply an absolute value. However, ‘strain’ on animals (AniPA) or ‘harm’ caused to plants and animals (n-HGTE) must be justified by overriding legitimate interests (in the following, ‘strain’ and ‘harm’ are combined in the single term ‘strain’). The question of whether strain can be justified in a specific case is answered by carrying out a weighing of interests. If justification is possible, the dignity of living beings/dignity of the animal is respected in the individual case despite the intervention; the proposed intervention can be carried out. If the strain cannot be justified by overriding legitimate interests, the dignity of living beings/dignity of the animal is not respected, and the proposed intervention must not be carried out.

Because in the context of the AniPA the weighing of interests as presented here is relevant only in connection with vertebrates, the term ‘dignity of living beings’ is not used in the following; ‘dignity of the animal’ can suffice.

## 3. Weighing of Interests

Putting the dignity of the animal into practice is a task primarily entrusted to people with a scientific background (e.g., people working in cantonal animal welfare enforcement, people conducting animal experiments). A major difficulty they face in carrying out the weighing of interests from the perspective of dignity is that it requires them to make a moral value judgment, i.e., a judgment that:

- is normative, not empirical (a normative judgment concerns the way something should be, not the way it is);
- consequently, cannot be arrived at and verified by an appropriate scientific method;
- is based on non-quantifiable criteria;
- allows the person making the judgment a degree of discretion, but without being random.

The weighing of interests is not an empirical method but a normative procedure. It is about comparing the value of different interests. The purpose of the comparison is to identify which interests carry the greater moral weight, i.e., are of a higher order and are therefore morally more significant. Consequently, it is not about describing or explaining an empirical situation or verifying a hypothesis. Rather, it is about substantiating a judgment based on moral considerations. However, empirical facts do play an important role: which animals are involved? What type of intervention is proposed? What effects would the intervention have on the individual’s ability to lead a species-specific life? Empirical scientific knowledge is therefore vital in order to conduct a weighing of interests. However, this knowledge is not enough to arrive at a well-founded moral judgment. It requires additional normative criteria which allow a weighing to be assigned to the interests for moral consideration on both the ‘strain’ side and the ‘interests’ side of the weighing of interests. Therefore, research scientists or those responsible for animal welfare implementation cannot undertake the necessary weighing of interests (solely) from a scientific perspective. They need to expand their outlook and consider how an intervention’s effects on the animals compare, from a moral perspective, with the interests described by the law as legitimate.

A request for the weighing of interests to be carried out presupposes that it is an open question whether or not a planned intervention respects an animal’s dignity. However, there are cases in which it is clear from the outset that dignity is not respected. Here, the legislature has conducted an abstract weighing of interests (in anticipation of individual procedures) and imposed a general ban on the intervention in question.

Animal welfare legislation contains several provisions in this regard. For example, Article 4 of AniPA prohibits the mishandling, neglect or unnecessary overworking of animals. In addition, Articles 16 to 24 of the Swiss Animal Protection Ordinance (AniPO) [4] list a large number of prohibited procedures. Further restrictions concerning breeding of animals and animal experiments are found in Articles 25 and 138, respectively, of the AniPO.

According to Article 8 of the n-HGTE, whether the dignity of the animal is respected must be determined on a case-by-case basis by carrying out a weighing of interests. The animal welfare legislation lacks such an explicit reference to a case-by-case basis, but the wording of the definition of dignity in Article 3(a) of the AniPA does focus on the individual. It makes little sense to assess issues such as castration or breeding of impaired animals across the board. A given intervention may entail different strains on the one hand and different legitimate interests on the other hand, depending on the particular case. For example, the strain caused to an animal by neutering should be assessed differently depending on the animal’s species, gender and age, and on the neutering method used. In addition, different legitimate interests of varying importance come into play depending on whether the animal is a cat, a horse, or a piglet.

The DSG of the FSVO has come up with a model procedure for the weighing of interests in context with the animal [5]. The weighing of interests is performed in seven steps:

### 3.1. Describing the Aim

The procedure starts by stating the aim of the proposed action and is based on the assumption that this action needs a normative exploration guided by the dignity concept. The aim should be framed as precisely as possible. What is the goal of the intervention considered? This question is not trivial but vital in order to assess the intervention (see Steps 3 and 4 in particular).

### 3.2. Presentation of the Facts

An accurate presentation of the facts is important in order to conduct the weighing of interests correctly. The facts include everything potentially of relevance in assessing the intervention at issue, that is, facts relevant with regard to the strains named in the dignity definition and to the interests involved. Knowing these facts is a prerequisite to identify and assess the strains concerned and the interests involved (see Section 3.5 and Section 3.6). If the weighing of interests is conducted without knowledge of the full facts, it may lead to an incorrect assessment.

### 3.3. Question of Suitability

The next step is to consider whether the intervention at issue is appropriate in order to achieve the intended aim in full, or at least in part. If this is the case, the weighing of interests is continued; the same applies in cases where it is not possible to gauge whether the intended aim will be achieved.

If it is clear from the outset that the intended aim cannot be achieved by means of the proposed intervention, the intervention must not be carried out and there is no need for finalizing the weighing of interests.

### 3.4. Question of Necessity

The necessity of an intervention is established if the intended aim cannot be achieved by a measure that entails no strain for the animal, or less strain than the proposed intervention. The question therefore arises of whether an alternative to the proposed intervention exists which could also be used to achieve the intended aim but imposes little or no strain on the animal (in this case, dignity would not be affected, for example: certain animal experiments can be replaced by experiments using cell cultures or by computer models).

If this is not the case, we need to clarify the extent to which a possible alternative would affect the animal’s dignity and whether it is gentler than the procedure originally proposed (example: castration of male piglets may no longer be carried out without anaesthesia). If an alternative is available, it is included in the weighing of interests and compared with the proposed intervention.

If the weighing of interests finds that the alternative respects the animal’s dignity, preference must be given to the alternative. However, the following must be borne in mind: when considering a particular intervention, there will often be alternatives which can indeed be used to achieve the intended aim and entail less strain for the animal but are more difficult or more costly to implement (e.g., more labour-intensive or more expensive) than the intervention being considered. The question arises of when the use of the alternative will be imposed instead of the intervention originally considered. This question should be assessed from the perspective of reasonableness. The greater the strain imposed by the original procedure, the greater the effort or expense the stakeholders can reasonably be expected to accept in relation to the alternative. 

### 3.5. Identification and Assessment of Strain

Based on full knowledge of the facts, the strain can be identified and weighed.

#### 3.5.1. Forms of Strain

For the purposes of animal welfare legislation, the forms of strain contained in the definition of ‘dignity’ (Article 3(a) of the AniPA) are crucial. These are:

- Causing pain, suffering or harm, exposing to anxiety
- Humiliation
- Major interference with the appearance
- Major interference with the abilities
- Excessive instrumentalization

In the context of the n-HGTE, the weighing of interests under Article 8 must focus in particular on substantial harm to species-specific properties, functions, or habits. The term ‘species-specific properties, functions or habits’ refers to the concept of what constitutes a normal life of a (free-living) member of a species under ideal environmental conditions. It also specifies and defines what is understood by ‘inherent good’, implying that it is appropriate for individual living beings to lead a life typical of their species. This stipulates that beings with an inherent good possess an “inherent worth” and hence dignity, for the sake of which they are to be respected. 

Species specificity includes particular natural development (growth), reproduction and associated behaviour, the specific type of food and food intake, specific behavioural patterns in social species as well as in non-social species, specific ability to adapt to new environmental conditions, and particular circumstances that typically cause unpleasant or pleasant sensations (pain and pleasure). However, we have to deal with grey areas where it is not always clear whether we have already gone beyond what is ‘normal’ in manipulating an animal’s environment.

In order to implement the requirements of the AniPA as well as those of the n-HGTE, the following forms of strain have been incorporated into the model procedure for the weighing of interests:

- Pain, suffering, anxiety
- Harm, especially impairment of growth, reproductive capacity, adaptive capacity, mobility, species-specific social behaviours
- Major interference with appearance
- Humiliation, excessive instrumentalisation
- Other

A definition of these terms is proposed in the document “Dignity of the Animal—Explanatory Notes on the Weighing of Interests” [5].

For the weighing of interests, the forms of strain identified must not only be allocated to the set criteria, but also be described in more detail.

#### 3.5.2. Assessment of Strain

Regarding the importance of the individual criteria mentioned above, or components of the inherent good of an animal, two points are crucial:

- Any change beyond what is normal must be taken into consideration if it represents a type of strain.
- Different degrees of strain should be distinguished.

Based on the ‘degree of severity’ classification used in animal experiments, a three-stage weighing system has been adopted:

* slight/mild strain
** moderate/substantial strain
*** severe strain

The weighing classes are categories. This is not a graduated but a lexicographic classification. The number of stars assigned to a category is therefore to be understood as descriptive, rather than quantitative. This is why the results for different categories cannot be added or totaled up.

What does it mean for the weighing if several types of strain are present? Crucially, the individual strains allocated to a weighing category cannot add up to a total that might lead to classification in a higher category. For example, if there is a mild degree of strain on several criteria (pain, suffering, anxiety, etc.), this does not mean that the overall strain becomes substantial or severe. Conversely, it also follows that, even if there is considerable strain across all criteria, it should be regarded as less important than a severe strain on one criterion only. The crucial factor in determining the overall weighing is therefore the greatest individual strain in each case. This does not mean that the number of affected criteria does not play a role in the weighing. Assuming that Case A involves a substantial impairment of mobility (two stars), while Case B also involves substantial suffering, the strain in Case B should be given more weight overall. Nevertheless, if the suffering in Case C is classified as severe, this strain must be given more weight than that in Case B, even if it is the only strain.

### 3.6. Identification and Assessment of Legitimate Interests

#### 3.6.1. Which Legitimate Interests Are Involved?

The weighing of interests compares the strains on the animals with other values and interests and must show whether the latter can be regarded as overriding. It is important to note that morally relevant assets are involved on both sides. The interests that are compared with the strains are not just any interests. This is expressed in the n-HGTE by the term ‘legitimate’. The non-exhaustive list given in Article 8 n-HGTE shows that the interests to be considered in this procedure must be important interests of society as a whole. The following are mentioned:

- human and animal health;
- guaranteeing food security;
- the reduction of harm caused to the environment;
- the preservation and improvement of environmental conditions;
- securing a substantial economic, social, or environmental benefit for society.

In the AniPA, the term ‘legitimate’ does not appear in connection with interests. Only in connection with animal experiments (Article 137 AniPO) is there an explicit reference to interests which may be used to justify experiments and should therefore be included in the weighing of interests. However, since this procedure in the context of animal welfare legislation also entails a moral value judgment, not just any interests can be presented either. Here too, the interests must merit the description ‘legitimate’. This is also confirmed by the quality of the interests mentioned in Article 137AniPO in connection with animal experiments:

- preservation or protection of the life and health of humans and animals;
- new knowledge on fundamental processes of life;
- protection of the natural environment.

Applied to animal welfare issues in general, the interests considered can also include interests protected by the Constitution. Individual interests can be considered if they are interests of a person as a citizen. This means interests in the sense of basic rights protected by the Constitution. However, individual interests in the sense of specific private interests cannot be included in the weighing of interests.

Because the same intention lies behind the legitimate interests referred to in the AniPA and the n-HGTE, the list of interests in Article 8 n-HGTE is adopted, with adaptations, for the weighing of interests in the context of animal welfare legislation. The following interests should be considered:

- human and/or animal health;
- expanding scientific knowledge;
- preservation and improvement of environmental conditions;
- protection against violation of fundamental rights such as economic freedom, freedom of ownership, freedom of research;
- other.

The final point (‘other’) takes account of the fact that there may be further interests to consider. However, they must be interests of society as a whole; private interests may not be listed here either.

As with the types of strain, the interests identified must not only be allocated to the set groups of interests) but also described in more detail.

#### 3.6.2. Assessment of Legitimate Interests

The list of interests in Article 8 n-HGTE does not have an order of priority intended by the legislature, i.e., they cannot be put into an absolute order such that, for example, human and animal health could be said to take precedence over the other interests. However, relative weighings are possible. For example, it can be said that human health is relatively important as a rule, while increasing knowledge has relatively minor importance. The ‘as a rule’ illustrates the importance of a case-by-case assessment, because the interests mentioned do not always carry the same weight. Regarding health, for example, the development of a treatment for a life-threatening disease would be given more weight than one for a non-life-threatening disease. The weighing of interests is always carried out on a case-by-case basis and there is a substantial degree of discretion.

For the assessment of legitimate interests, a four-stage weighing system (* to ****) was chosen, i.e., one weighing category more than for strain. Because strain has to be justified by overriding interests, this is necessary in order not to rule out three-star strains in advance. Again, the results in the different categories cannot be added or totaled up. The crucial factor in determining the overall weighing is therefore the most significant legitimate interest in each case.

### 3.7. Comparison: Strain vs. Legitimate Interests/Conclusion

In principle, the weighing of interests should include all strains and all legitimate interests involved in the individual case. However, the decisive factors are the severest form of strain and the most significant legitimate interest. Strain can be justified only by overriding legitimate interests. This means that dignity is only respected if the most significant legitimate interest can be given more weight than the severest form of strain. If the interest is given the same or less weight than the strain, the interest is indeed legitimate but not overriding. In this case, the strain cannot be justified and the dignity of the animal is not respected. 

## 4. The Dignity of Kangal Fish

The DSG uses the procedure for the weighing of interests described above to deal on behalf of the FSVO with specific questions concerning the dignity of the animal which are not a priori governed in the AniPA. In what follows, the weighing of interests is applied to the question whether the use of kangal fish for therapeutic and other purposes can be justified by overriding interests.

Kangal fish originate from Eurasia. They live in swarms on the ground of rivers, lakes, little ponds and muddy creeks, where they can hide among stones and in the underwater vegetation and where they feed on plant particles [6,7]. They can also be found in the hot springs of the Turkish spa town Kangal where the available natural food resources for the fish are scarce. These spas are visited by patients suffering from chronic skin diseases like psoriasis and atopic eczema [8]. The hungry fish nibble on the skin of the affected body parts (e.g., legs, hands) of the patients, hereby removing necrotic skin particles. This may result in a mitigation of the itch which can be extreme in the case of these diseases [9]. By now, kangal fish are also used in Switzerland [10] and other European countries in the therapy of chronic skin diseases. Furthermore, a trend has developed to use kangal fish not only for therapeutic applications but also for cosmetic or recreational purposes (e.g., in nail studios, spas, and bars). In Switzerland, an authorization is required for the keeping and breeding of wild animals, including fish, on a commercial basis. As far as the therapeutic use of kangal fish is concerned, the authorization has been granted on specific conditions by the cantonal veterinary authorities. 

Eventually, the veterinary office of a canton was confronted with the request of a bar owner who wanted to keep kangal fish in a footbath that could be used by his guests while sitting at the bar. The cantonal office forwarded the request to the FSVO and the DSG was assigned to assess the question whether the use of kangal fish for therapeutic and other purposes can be justified by overriding interests. To this aim, the model procedure for the weighing of interests established by the DSG was applied.

### 4.1. Weighing of Interests for Therapeutic Use 

The fish are kept in an indoor aquarium which is furnished (e.g., with stones, water plants) to provide the fish with an adequately enriched environment and transferred to the therapy basin for the time of the treatment. The feed is restricted permanently in order to assure that they eat the skin particles of the patients. For treatment, the patients dip the affected body parts in the therapy basin. After the treatment, the fish are transferred back to their home aquarium. 

The first question is whether the use of kangal fish is suitable for attaining the intended goal, namely, to mitigate the severe itch associated with chronic skin diseases. This seems to be the case, at least to some extent [9]. Likewise, the question of whether the use of the kangal fish is necessary can be answered with yes. Many of these patients have suffered from these diseases for years and have undergone various treatments without success. For them, the kangal fish therapy can be a straw to clutch at. 

When used as therapy animals, several forms of strain are imposed on the kangal fish. Stress is supposed to be an important issue in fish welfare [11,12]. Kangal fish used as therapy animals may be stressed by the barren environment in the therapy basin, where they have no possibility to hide, and by frequent handling due to the transfer from the home aquarium to the therapy basin and back. Additionally, their social structure is disturbed because of the frequent transfers and they are hungry at least periodically. However, there is no evidence of substantial risk of serious injuries, diseases or premature death for these fish. Furthermore, they are excessively instrumentalised by the fact that they are perceived as medicament without respecting their needs.

In total, the DSG assessed the strain imposed on the kangal fish used in therapy as substantial (two stars, the second of three weighing categories). 

Considering the interests, the health of the patients and their craving for relief from their severe itch were found to be of foremost concern. The DSG accorded three stars to this interest, that is, the group attached more weight on the interest than on the strain imposed on the fish. Since there is an overriding interest in the case of the therapeutic use of kangal fish, the strain for the fish can be justified and therefore, their dignity is respected. 

### 4.2. Weighing of Interests for Cosmetic or Recreational Use

At large, the housing and handling of the fish in the case of cosmetic or recreational use is similar to the therapeutic use, probably with the exception that the fish will remain for a longer time in the treatment basin. As with the therapeutic use, kangal fish seem appropriate to attain the intended goal; namely, to remove skin particles from the feet of nail studio clients or to provide some fun for bar visitors by being tickled by the fish, and the like. Hence, we can affirm the suitability. Considering the necessity, we look for alternatives to the use of kangal fish for cosmetic or recreational purposes which do not depend on animals. Because there are many conceivable alternatives which may fulfill the same intentions without the employment of animals, the necessity to use kangal fish is not given and cannot be justified. Thus, kangal fish must not be used for cosmetic or recreational purposes and there is no need for finalizing the weighing of interests.

The DSG stipulated that the dignity of the kangal fish is respected when used for therapeutic purposes because there is the overriding interest of patients with chronic skin diseases which justifies the strain imposed on the fish. On the other hand, the DSG considered the use of kangal fish for cosmetic or recreational purposes a disregard of the dignity of the fish because, in this case, the question of necessity was denied. The DSG concluded, therefore, that the authorization for the keeping and breeding of fish on a commercial basis should be granted for the therapeutic use of kangal fish only. Subsequently, the application of the bar owner was not approved by the cantonal authority. The bar owner did not accept the denial and appealed to the regional court but was turned down by the court. In other words, the court supported the approach of the DSG.

The use of kangal fish is at issue in other cantons, too, and it is important that the question of authorization is dealt with consistently in all cantons. In order to harmonise the implementation concerning the use of kangal fish among cantons, the FSVO published expert information [13] which recommends the cantonal authorities to decline requests for the use of kangal fish for cosmetic or recreational purposes. To substantiate this recommendation, the FSVO refers to Article 3 (a) (definition of dignity) and Article 4 (2) of the AniPA and outlines that based on the weighing of interests, the use of kangal fish for such purposes was judged as a disregard of the dignity of the fish. This expert information is accessible on the FSVO homepage.

## 5. Conclusions

As demonstrated in the example of kangal fish used for therapeutic or recreational purposes, the proposed procedure for the weighing of interests is a valuable tool when it comes to applying the dignity concept in the context of the AniPA. However, there are several aspects in the procedure as well as in the dignity definition used in the AniPA that need further evaluation. This applies to some of the terms (e.g., humiliation) as well as to the question of which interests qualify to be considered in the weighing of interests. Furthermore, the question of how to weigh strains as well as interests is worth further debate.
